# Antagonistic interplay between pH and food resources affects copepod traits and performance in a year-round upwelling system

**DOI:** 10.1038/s41598-019-56621-6

**Published:** 2020-01-09

**Authors:** Victor M. Aguilera, Cristian A. Vargas, Hans G. Dam

**Affiliations:** 1 Centro de Estudios Avanzados en Zonas Áridas (CEAZA), Bernardo Ossandón #877, Coquimbo, Chile; 2 Facultad de Ciencias del Mar, Depto. Biología Marina, Universidad Católica del Norte, Coquimbo, Chile; 3 Instituto Milenio de Oceanografía, Universidad de Concepción, Concepción, Chile; 4 Aquatic Ecosystem Functioning Lab (LAFE), Department of Aquatic Systems, Faculty of Environmental Sciences and Environmental Sciences Center EULA Chile, Universidad de Concepción, Concepción, Chile; 5 Center for the Study of Multiple-drivers on Marine Socio-Ecological Systems (MUSELS), Universidad de Concepción, Concepción, Chile; 6 Department of Marine Sciences, University of Connecticut, 1080 Shennecossett Rd, Groton, CT 06340-6048 USA

**Keywords:** Carbon cycle, Ecophysiology, Marine chemistry

## Abstract

Linking pH/*p*CO_2_ natural variation to phenotypic traits and performance of foundational species provides essential information for assessing and predicting the impact of ocean acidification (OA) on marine ecosystems. Yet, evidence of such linkage for copepods, the most abundant metazoans in the oceans, remains scarce, particularly for naturally corrosive Eastern Boundary Upwelling systems (EBUs). This study assessed the relationship between pH levels and traits (body and egg size) and performance (ingestion rate (IR) and egg reproduction rate (EPR)) of the numerically dominant neritic copepod *Acartia tonsa*, in a year-round upwelling system of the northern (23° S) Humboldt EBUs. The study revealed decreases in chlorophyll (Chl) ingestion rate, egg production rate and egg size with decreasing pH as well as egg production efficiency, but the opposite for copepod body size. Further, ingestion rate increased hyperbolically with Chl, and saturated at ~1 µg Chl. L^−1^. Food resources categorized as high (H, >1 µg L^−1^) and low (L, <1 µg L^−1^) levels, and pH-values categorized as equivalent to present day (≤400 µatm *p*CO_2_, pH > 7.89) and future (>400 µatm *p*CO_2,_ pH < 7.89) were used to compare our observations to values globally employed to experimentally test copepod sensitivity to OA. A comparison (PERMANOVA) test with Chl/pH (2*2) design showed that partially overlapping OA levels expected for the year 2100 in other ocean regions, low-pH conditions in this system negatively impacted traits and performance associated with copepod fitness. However, interacting antagonistically with pH, food resource (Chl) maintained copepod production in spite of low pH levels. Thus, the deleterious effects of ocean acidification are modulated by resource availability in this system.

## Introduction

Anthropogenic CO_2_ emissions to the atmosphere since the industrial revolution have reduced the pH of the surface open ocean at a steady rate of 0.02 pH units per decade^1^, giving rise to an unprecedented ocean acidification (OA) process in millions of years^2^. The rapid progression of OA is challenging the adaptive potential of marine biodiversity and compromising the ecosystem services oceans provide to humans^3,4^. In particular, biologically productive Eastern Boundary Upwelling Systems (EBUs) are naturally low in pH^5^, but the synergy with OA has decreased pH levels below thresholds^5,6^ that impact the tolerance of the biota and threaten the social livelihood these globally relevant marine areas provide^7^. Within the urgent need for more and better worldwide observations of chemical parameters associated with OA^8^, physical-chemical assessments in upwelling systems^5,6,9^ have progressively incorporated the effect of carbonate chemistry on organismal physiology^10–13^. However, upwelling areas in the Humboldt EBUs remain understudied^14,15^, precluding their integration to regional and global analysis of ocean perturbations due to climate change^16^.

In addition to contemporary pH threshold levels, future changes in ocean chemistry due to OA will decrease pH levels below those observed during recent evolutionary history of species as well^17^. Laboratory experiments aiming to test species sensitivity under low pH (and very low) levels have thus far been a common way to assess the potential effects of OA on marine organisms^18,19^. Although pH conditions vary widely temporally within species habitats, with few exceptions^15,20–22^ this natural variation has largely been ignored in the design of experimental OA studies. Natural pH variation regulates phenotypic plasticity and adaptive potential of local populations^15^, and its omission in the design of OA experiments can lead to results which may not necessarily reflect future responses to global stressors^21^.

In upwelling areas, pH levels are highly variable both spatially and temporally^5,6,9,23^, already reaching and occasionally exceeding OA scenarios projected for open ocean areas^6,15,24^. Hence, ambient pH variation might constitute a relevant environmental factor affecting physiological processes of local populations such as ingestion and reproduction, which are critical to any autopoietic or organized living system. In the now large literature on studies of the effects of OA on marine biota^25,26^, studies on copepods are a minority. Yet, copepods are the most abundant metazoans in the oceans^27^, which play pivotal roles in pelagic trophodynamic^28^, biogeochemical cycles^29^ and ecosystem services^30^. The consensus from copepod studies is that adult stages are resilient to OA^31,32^. However, very few of these studies are from field observations. Thus, there is an urgent need for field studies that can verify or refute these laboratory studies. The neritic copepod *Acartia tonsa* (Copepoda, Calanoida) is among the most abundant and temporarily prevalent species in upwelling areas of the productive Humboldt EBUs, where it inhabits near surface waters^33,34^ and recruits continuously^33,35^ in neutral to acidic (i.e., low pH values)^36^, yet productive conditions.

In the present study we assessed the linkage between environmental (pH, temperature, oxygen, salinity, total alkalinity and chlorophyll-*a* (Chl)) and *A*. *tonsa*’s traits (body and egg size) and performance (ingestion and egg reproduction rate). The study was conducted in the coastal area of the Atacama Desert (23°S, Table 1), in the northern Humboldt EBUs, whose climatology is dominated by wind-driven upwelling^37,38^. Twelve (12) nearshore surveys were conducted during morning time between austral fall and early spring 2015 (May and September) in an advective environment affected by upwelling currents. Independent, uni- and multi-variate analysis revealed copepod traits and performance were significantly correlated with pH and Chl. Both food resources (Chl) and pH were factorized in “High” and “Low” conditions based on functional relationship (Chl) and present day and future *p*CO_2_ conditions, respectively. To contextualize current findings into the perspective of climate change effects in EBUs, pH levels were compared with laboratory OA experiments (*n* = 40) with copepods from different latitude and marine environments. Our results show that high Chl concentrations could alleviate the negative impact of naturally stressful low-pH conditions characteristic of upwelling environments on copepod traits and performance.

**Table 1:** Sampling depth, location, observation period, and physical/chemical measurements (*) and biological estimates (^+^).

Site depth (m)	Location	Sampling year	Sampling period	Environmental* Biological^+^ Variables	Sampling depth (m)
40	23°27′S	2015	May to	CTDO-casts*	0–27
	70°37′W		September	pH and A_T_* Chlorophyll-a* Body size^+^ Ingestion rates^+^ Egg production rates^+^	10 10 20–15 20–15 20–15
				Egg size^+^	20–15

## Results

**Figure 1:**
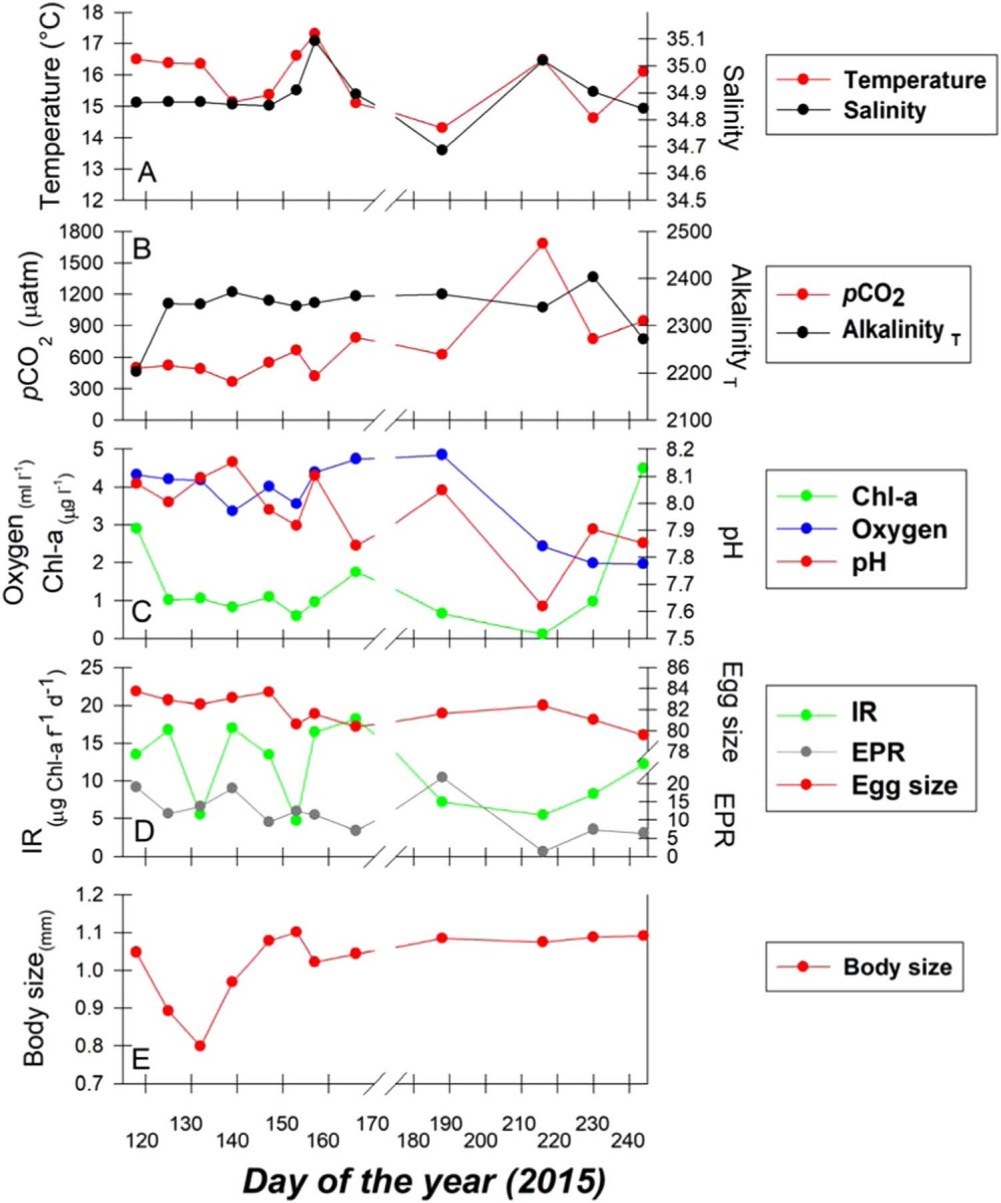
Temporal display of physical-chemical (panels A to C) and traits/performance of wild caught *A*. *tonsa* females (panels D to E) data versus day of the year. The break in the X-axis denotes a change in sampling frequency from 7 ± 2 (*n* = 7) to 20 ± 7 (*n* = 4) days.

**Figure 2:**
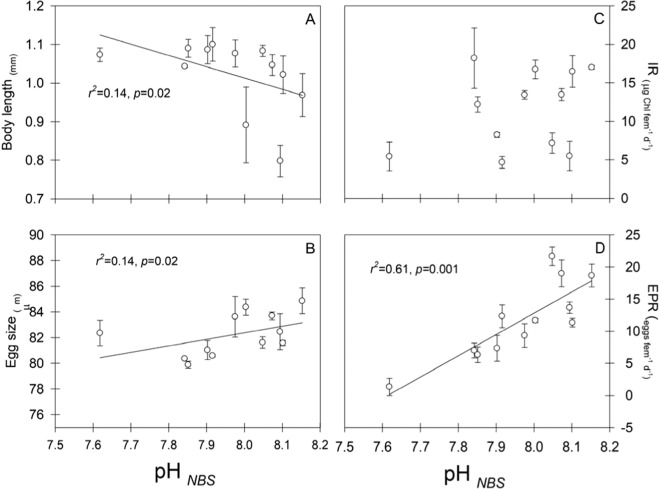
Single correlations between mean pH and copepod traits (**A**,**B**) and performance (**C**,**D**). Average value (±SE) of twelve (12) observations of body (**A**) and egg size (**B**), and ingestion rate (IR) (**C**) and egg production rate (EPR) (**D**) of adult *A*. *tonsa* females versus seawater pH recorded at 10 m depth. Shown within each panel are the coefficients of determination of the regression, r^2^, and the *p*-value for the regression throughout a five month period.

Variations of environmental parameters (pH, temperature, oxygen, salinity, total alkalinity and chlorophyll-*a*) and biological (body length, ingestion rate, egg reproduction rate and egg size) variables observed during twelve (12) oceanographic cruises carried out in this year-round upwelling system of the Humboldt EBUs are shown in Fig. 1. Single linear regressions of environmental versus biological data revealed pH, expressed in the NBS scale (National Bureau of Standards scale) was negatively related to copepod body size (y = 3.33–0.29*x, r^2^ = 0.14, *p* = 0.02, *n* = 36), but positively related to egg production rate (EPR) (y = −252.96 + 33.22*x, r^2^ = 0.61, *p* = 0.001, *n* = 36) and egg size (y = 41.34 + 5.13*x, r^2^ = 0.14, *p* = 0.02, *n* = 36) (Fig. 2). Ingestion rate was not correlated with pH (*p* = 0.05, Fig. 2). There were, however, no significant correlations between copepod body length and egg production (*p* = 0.46) or egg size (*p* = 0.06), nor between egg production and egg size (*p* = 0.16). Biological-environmental relations were independently evaluated through a Distance based Linear Model (DistLM) and a Principal Coordinate (PCO) analysis, which scores (adj. R^2^) step-wise relations occurring on a multidimensional space and reduces it to the two most significant planes of variability, respectively. Accounting for much (>62%) of biological variance, DistLM (Table 2) and PCO (S.I. Figure 1) supported the significant but opposite effect between pH and Chl on copepod traits and performance.

**Table 2:** Identification of relevant environmental drivers explaining changes in performance and traits of *A*. *tonsa* according to the distance based Linear Model. The model was run among Euclidean and Similarity matrices applying a stepwise selection procedure and sequential R^2^ (Adj.) selection criteria.

Variable	Adj. R^2^	SS (trace)	Pseud-F	P-value	Prop.	Cumul.	res. d.f.
+pH	0.44	3672	29	0.0001	0.49	0.46	34
+Chl	0.48	481	3.4	0.041	6.37E-2	0.51	33
+Sal	0.50	231	2.2	0.121	3.06E-2	0.54	32
+Temp	0.50	175	1.2	0.29	2.31E-2	0.56	31
+Oxy	0.52	246	2.4	0.10	3.21E-2	0.59	30

**Figure 3:**
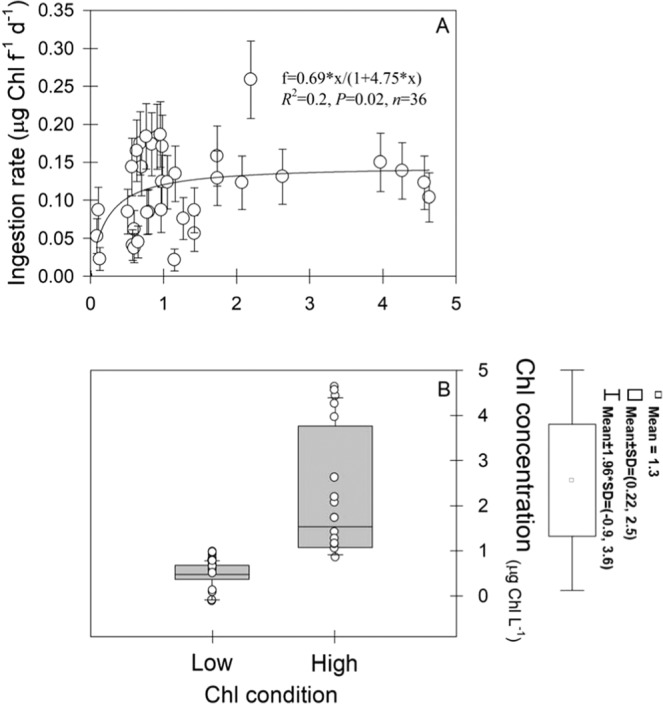
Chlorophyll (Chl) categorization in high and low levels. Significant Chl/ingestion rate (IR) relationship was assessed through a functional non-linear regression which showed 1 µg Chl L^−1^ significantly delimited (F_1,35_ = 6.43, *P*-value = 0.02, *n* = 36) the transition between the ascending and stable IR curve (A). Relative contribution observed at 10 m depth was 41.7% (H) and 58.3% **(**L).

**Figure 4:**
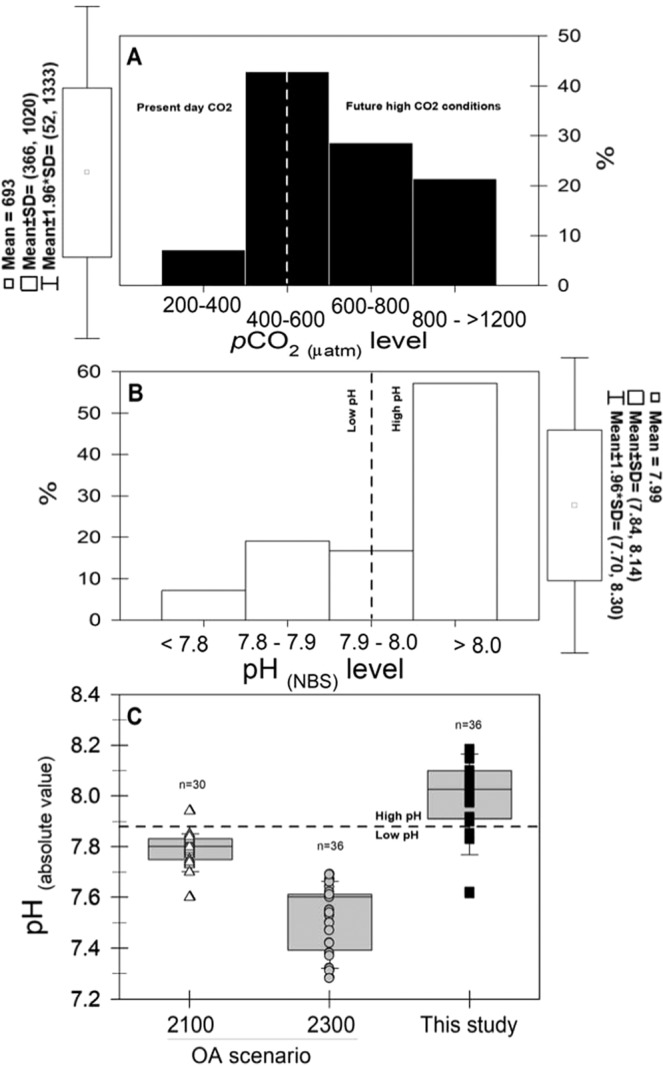
Factorization of upwelling pH levels (High and Low). The threshold between present day and future *p*CO_2_ levels segregated on *in situ p*CO_2_ estimations (**A**), the equivalent pH levels representing high (>7.89) and low (<7.89) levels (**B**). Upwelling pH values were compared with values globally considered in laboratory studies (*n* = 40) assessing copepod sensitivity to OA conditions expected by the years 2100 and 2300 (**C**).

**Figure 5:**
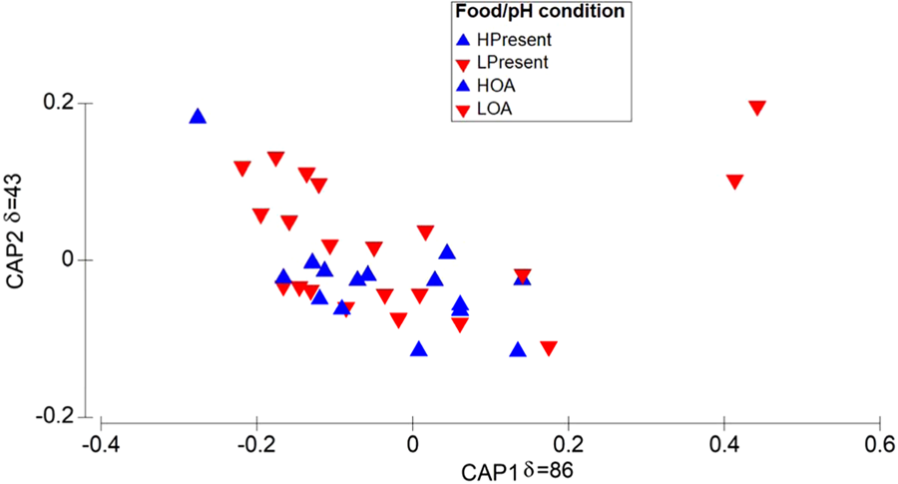
Allocation of copepod body size, EPR and egg size according PERMANOVA CAP 1 and 2. CAP 1 clearly segregated high (grey color) from low (black) pH effect on copepod traits and performance. Under low pH conditions, CAP 2 separated high (blue) from low (red) Chl effects.

To further explore this likely antagonistic interaction^39^ between food resource and pH, observed Chl and pH were categorized as follow. Based upon a functional non-linear relationship (r^2^ = 0.16, *P* = 0.02, *n* = 36, y = 0.0146*x/(0.2106 + x)) between Chl and copepod ingestion rate (IR) (Fig. 3A), which yielded a saturation concentration of ~ 1 µg Chl L^−1^, and the analysis of Chl distribution (Fig. 3B), Chl concentration was grouped in either high (H > 1 µg Chl L^−1^) or low (L < 1 µg Chl L^−1^) conditions. The relative contribution of high (H) and low (L) levels was 41.7% and 58.3%, respectively (Fig. 3B). The threshold between present day (≤400 µatm) and future (>400 µatm) *p*CO_2_ ocean levels was superimposed on *p*CO_2_ values estimated at 10 m depth at the upwelling site (Fig. 4A), and the equivalent pH levels represented “High” (present day, pH > 7.89) and “Low” (future, pH < 7.89) pH-conditions (Fig. 4B). Categorized pH conditions were compared with experimental levels considered in multiple (*n* = 40) studies aiming to evaluate the impact of ocean acidification (OA) on copepod performance, including species other than *Acartia tonsa* (Fig. 4C). Table 3 summarizes location, species, mean experimental pH levels, and pH measurement methodology of forty studies addressing performance effects on pelagic marine copepods due to OA scenarios projected for the year 2100 and 2300^40^. The results of statistical comparison are shown in Table 4. Although pH values for this study were significantly higher than both OA scenarios, low pH values observed occasionally at 10 m depth at the study site partially overlapped those of the 2100 OA condition. After confirming the assumption of homogeneity of multivariate dispersions (PERMDISP test), and 999 permutations of residuals under a reduced model, the 2 × 2 PERMANOVA analysis indicated there were significant differences in copepod reproduction between H and L treatments of pH Factor at low Food conditions (Pseud-F_1,35_ = 27, *P* = 0.0001). However, at high Food conditions, copepod reproduction was similar at H and L pH levels. Seawater pH accounted for a relatively higher component of variation (sq. root = 14.5) than Chl (sq. root = 9.3) or the interaction between both factors (sq. root = 9.7), and significantly lower physiological rates were observed under low pH ( = OA) conditions. Chl concentration did not affect copepod EPR under high pH levels while under low pH conditions, copepods exposed to high Chl levels showed significantly higher EPR. This is graphically shown by the Canonical Analysis of Principal Coordinates (CAP), which was conducted after the PERMANOVA comparison (Fig. 5). Indicative of the relative high strength of the correlation between biological data and food/pH group differences, the sizes of CAP 1 and 2 were δ = 0.86 and δ = 0.43, respectively, significantly segregating (pH: Pseud-F_1,35_ = 7, *P* = 0.001) high and low pH conditions (CAP 1 axis). In spite of subtle dispersion (one sampling day), most of the high food data tend to overlay in the same plane than low pH conditions across the CAP 2.

**Table 3:** Summary of laboratory experiments with different copepod species under pH levels associated to OA- (*n* = 40), whose species names were checked in the World Register of Marine Species. Scale of pH values not informed (N.I.).

Lat.	*Species names*	pH OA–scenario		Scale	Source
		2100	2300		
34 N	*Acartia steueri* Smirnov, 1936	—	7.55 ± 0.01	NBS	Kurihara *et al*., 2004
34 N	*Acartia tsuensis* Ito, 1956	—	7.32	NBS	Kurihara & Ishimatsu, 2008
24 N	*Acartia pacifica mertoni* Steuer, 1917, *Centropages tenuiremis* Thompson & Scott, 1903	7.85 ± 0.04	7.37 ± 0.08	NBS	Zhang *et al*., 2011
57 N	*Calanus finmarchicus* Gunnerus, 1770	7.77 ± 0.04	7.37 ± 0.02	NBS	Mayor *et al*., 2012
78 N	*Calanus glacialis* Jaschnov, 1955	7.6		NBS	Weydmann *et al*., 2012
77 N	*Acartia sp*.		7.53	N.I.	Vehmaa *et al*., 2012
54 N	*Acartia tonsa* Dana, 1849	7.94 ± 0.08	—	NBS	Rossoll *et al*., 2012
49.9	*Tisbe battagliai* Volkmann-Rocco, 1972		7.67 ± 0.21	N.I.	Fitzer *et al*., 2012
24 N	*C*. *tenuiremis*	7.83 ± 0.02	—	NBS	Li & Gao, 2012
59 N	*Acartia bifilosa* Giesbrecht, 1881	—	7.6	N.I.	Vehmaa *et al*., 2013
63 N	*C*. *finmarchicus*		7.31 ± 0.04	NBS	Pedersen *et al*., 2013
50 N	*Centropages typicus* Krøyer, 1849, *Temora longicornis* Müller, 1785	7.85 ± 0.02	7.78 ± 0.02	NBS	McConville *et al*., 2013
78 N	*C*. *glacialis*, *Calanus hyperboreous* Krøyer, 1838, *Oithona similis* Claus, 1866	7.80 ± 0.05	7.6 ± 0.09	Total	Lewis *et al*., 2013
38 N	*Acartia clausi* Giesbrecht, 1889	7.83 ± 0.02	—	Total	Zervoudaki *et al*., 2014
59 N	*Eurytemora affinis* Poppe, 1880	—	7.28	N.I.	Almén *et al*., 2014
63 N	*C*. *finmarchicus*	—	7.64 ± 0.02	Total	Pedersen *et al*., 2014
59 N	*A*. *bifilosa*	—	7.6	NBS	Engström-Öst *et al*., 2014
79 N	*C*. *glacialis*, *C*. *hyperboreous*		7.37	Free	Hildebrandt *et al*., 2014
58 N	*A*. *tonsa*	7.82 ± 0.05	7.61 ± 0.06	NBS	Cripps *et al*., 2014
59 N	*A*. *bifilosa*	—	7.5	Total	Vehmaa *et al*., 2015
23 N	*Acartia grani* Thompson & Scott, 1903	7.81 ± 0.04	7.62 ± 0.03	NBS	Isari *et al*., 2015
79 N	*Pseudocalanus acuspes* Giesbrecht, 1881	7.8 ± 0.05	7.61 ± 0.07	Total	Thor & Oliva, 2015
58 N	*P*. *acuspes*	7.7 ± 0.07	7.47 ± 0.07	Total	Thor & Oliva, 2015
24 N	*A*. *pacifica*	7.79 ± 0.02	—	NBS	Jin *et al*., 2015
23 N	*A*. *grani*, *Oithona davisae* Ferrari & Orsi, 1984	2300	7.66 ± 0.01	Total	Isari *et al*., 2015
37 N	*Tigriopus japonicus* Mori, 1938	7.79 ± 0.01	7.61 ± 0.02	N.I.	Oh *et al*., 2017
58 N	*P*. *acuspes*	7.8 ± 0.05	7.61 ± 0.07	Total	De Wit *et al*., 2015
58 N	*P*. *acuspes*	7.75 ± 0.02	7.54 ± 0.08	Total	Thor & Dupont, 2015
42 N	*A*. *clausi*, *C*. *typicus*	7.83 ± 0.01	7.74 ± 0.01	Total	Zervoudaki *et al*., 2017
80 N	*C*. *glacialis*	—	7.70 ± 0.03	Total	Thor *et al*., 2016
59 N	*C*. *glacialis*	7.69 ± 0.01	7.47 ± 0.01	Total	Bailey *et al*., 2017
60 N	*C*. *finmarchicus*	7.61 ± 0.09	7.42 ± 0.04	Total	Runge *et al*., 2016
80 N	*C*. *glacialis*	7.69 ± 0.01	7.47 ± 0.01	Total	Bailey *et al*., 2017
59 N	*E*. *affinis*	7.74 ± 0.05	7.67 ± 0.06	Total	Almén *et al*., 2016
39.5 S	*A*. *tonsa*	—	7.58 ± 0.03	NBS	Aguilera *et al*., 2016
24 N	*Calanus sinicus* Brodsky, 1962	7.84 ± 0.06	7.42 ± 0.09	NBS	Zhang *et al*., 2016
37 N	*C*. *sinicus*	—	7.70 ± 0.03	NBS	Kang *et al*., 2016
79 N	*C*. *glacialis*	—	7.62 ± 0.02	Total	Hildebrandt *et al*., 2016
79 N	*C*. *glacialis*	—	7.48 ± 0.1	Total	Thor *et al*., 2018
58 N	*P*. *acuspes*	—	7.67 ± 0.04	Total	Almén *et al*., 2017

**Table 4:** Metrics of categorized pH levels and multiple comparisons of *p*-values under Kruskal-Wallis test (H_2, 102_ = 78, *P* = 0.001). Significant differences after multiple comparisons of *p* values are denoted as *.

Factor	Level	*N*	Mean	Std. Dev.	Median	*p*-values 2-tailed
pH	2300	36	7.53	0.13	7.60	*
	2100	36	7.79	0.15	8.02	*
	This study	30	7.99	0.06	7.80	*

**Figure 6:**
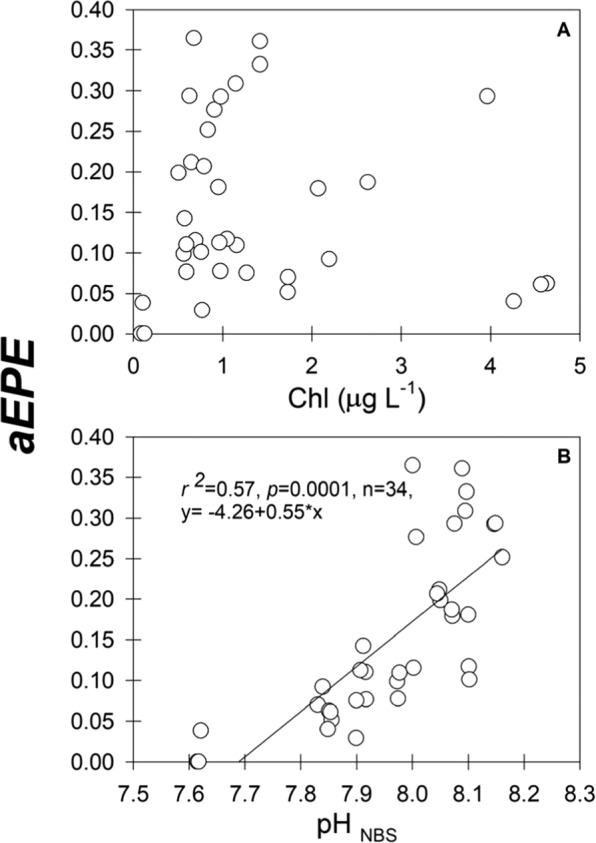
Relationships between with autotrophic egg production efficiency (aEPE) and pH (**A**) and food resources (i.e., Chl) (**B**). The aEPE is the ratio between the weight-specific EPR/IR.

The autotrophic egg production efficiency (aEPE), calculated as the carbon-based ratio of egg production and ingestion rate (EPR/IR) was analyzed through linear regressions against Chl and pH (Fig. 6). Food resource did not affect aEPE, but there was a significant positive (r^2^ = 0.57, *p* = 0.001, *n* = 34) relation with pH, and a significant, but negative (y = 0.28–0.04*x, r^2^ = 0.1, *p* = 0.049, *n* = 34) relation to O_2_ concentration (figure not shown).

## Discussion

This study showed a link between variations in pH and copepod traits and performance in the upwelling system of the Humboldt Eastern Boundary Upwelling system (EBUs). In particular, deleterious effects of low pH on EPR, egg size, and egg production efficiency were evident after independent statistical analyses of the observations. Moreover, the deleterious effects of low pH effects on EPR were mitigated by the availability of chlorophyll, suggesting that the effects of pH on copepod performance in this system are modulated by food resource. These findings have implications for our understanding of organismal response to OA.

We first consider the results of this study within the context of the study site. The coastal hydrography in the arid region of northern Chile is under the permanent influence of upwelling favorable winds, which show little seasonal variation^37,38^. In agreement with previous studies^36^, during the five months of observations at the 10 m depth of our study, intense upwelling episodes characterized by cold (<14 °C), oxygen deficient (<2 mL L^−1^), and low pH (<7.89_NBS_) water accounted for 31% of the cruises. Further, extremely low pH Equatorial Sub Surface Water (ESSW) is often upwelled into the photic zone^36,41^. The copepod *Acartia tonsa*, which is distributed in the uppermost 40 m of the water column in this system^33^, exploits the chlorophyll maximum that is typically found between 4 and 10 m^29^, and experiences generation times of two weeks to three months in this system^33^, depending on the prevailing temperature and food availability, without any obvious phenological cycles. A decrease in reproductive traits and performance (egg size, EPR and aEPE) of *A*. *tonsa* was evident when low-pH water prevailed (Fig. 2 and Table 2). Importantly, these observations cannot be explained by changes in body size since this variable behaved exactly the opposite in response to pH (Fig. 2). Either through behavioral migration or forced by advection into deeper waters^33^, *A*. *tonsa* females in this system are exposed to even lower pH waters than reported here. This exposure to a broad range of pH values can yield specialist/generalists distribution vs reproduction trade-offs^20,21,42–44^ and underlie population or species-specific differences in the habitat use. For example, Lewis *et al*.^24^ showed that surface-restricted *Oithona similis* responded negatively to experimental pH manipulations resembling deep high *p*CO_2_/low pH water found in the Arctic Ocean. Similarly, Aguilera *et al*.^45^ showed the reproduction of coastal *A*. *tonsa* individuals was associated with river-induced low pH water, and that females exhibiting a narrow and temporarily stable environmental pH variation were more stressed under experimental low-pH conditions associated with OA than estuarine counterparts exposed to wider and more fluctuating environmental pH variations^44^. Here, we found that reproductive females likely belonging to different cohorts of a population showing several production events per year^31,33^, are at times already experiencing in their habitat the negative impacts of pH levels which were expected for the year 2100 in other ocean regions (Fig. 4). Although with low frequency, the negative impact of surface irruptions of deep low-pH upwelled waters on neritic plankton should be concerning given the expected increase in upwelling intensity in some EBUs^46^, which can modify upwelling frequency and magnitude^23^.

Along with temporally variable heterotrophic components^29,47^, chlorophyll-*a* is a valuable index of phytoplankton upon which *A*. *tonsa* efficiently preys^48^ to maintain continuous reproductive output^49^. In this highly productive coastal upwelling system (gross primary production = 20 g C m^−2^ d^−1^ ^50^) the contribution to the secondary production and biomass of microzooplankton like ciliates is relatively low (<100 mg C m^−2^ d^−1^) for the upper 40 m depth^47^. Further, previous studies have shown the temporal dominance of small sized diatoms on the diet of *A*. *tonsa*^48,49^. Thus, in this system *A*. *tonsa* likely derives the bulk of its carbon ration from an herbivorous diet. The phytoplankton biomass (Chl) standing stock is constrained to a very narrow continental shelf (<20 km) in EBUs^51^ due to wind-driven Ekman divergence, leading to a persistent offshore reduction of food resources for plankton populations, which is exacerbated in the study area due to the occurrence of upwelling “shadows”^52^. Focused on pelagic communities inhabiting nearshore waters exposed directly to upwelling filaments and meandering currents^53^, this study was conducted in a section where Chl standing stock is relatively lower, but temporarily more stable. The bi-dimensional ordination (CAP 1&2) performed after the factorial Chl/pH PERMANOVA test suggests that Chl levels exerted a significant positive effect on copepod performance at low pH levels. Therefore, when phytoplankton biomass was high copepod reproduction reached roughly similar levels as observed at higher pH levels (Fig. 5). Indeed, unrelated to changes in food availability, the egg production efficiency (aEPE) was negatively affected by low pH levels (Fig. 6). This observation might reflect resource reallocation under stressful and energy demanding low-pH conditions^18,44,54,55^. Unlike larger, cold-water copepod such as *Calanus* sp., adult *A*. *tonsa* females do not store lipids^56^, and their egg production reflects food consumption within the previous 24 h^57,58^. This is further supported by the observed decrease in egg size, which is related to yolk availability to cover energetic requirement during early development^56^. The mitigating effect of high food availability to the deleterious effects of pH on the reproductive performance of *A*. *tonsa* might help explain its year-round prevalence in corrosive, but productive upwelling systems^15,36^. This food by pH interaction might also explain why the majority of laboratory experiments, which are done under food replete conditions, do not detect effects of low pH on copepod performance. More importantly, it suggests that cost of exposure to low pH is resource dependent. Thus, resource availability should be considered as a variable in studies of the response of the biota to global change.

More intense winds in EBUs associated with CO_2_–driven climate perturbations suggest more advection and less primary production in the coastal edge^51^, and more frequent/intense upwelling episodes^6,9,46^. Hence, food availability and pH levels might be critical environmental drivers for local pelagic populations. The zooplankton community, which is heavily dominated by copepods in this system, efficiently channels phytoplankton production to either anchovy or sardine fisheries, which places the Humboldt EBUs among the most productive EBUs^59^. However, the highly productive Peru–Chile upwelling system within the Humboldt EBUs currently experiences two major stressors–the world’s largest Oxygen Minimum Zone and CO_2_-oversaturated upwelling areas, with potential negative consequences for the biological performance of pelagic populations^60^, the carbon cycle, climate regulation, and global food supply^7,59^. This study provides standardized new data of carbonate system parameters and its relationship with the traits and performance of a dominant copepod species, which may represent zooplankton responses to current and future pH-conditions in an important upwelling ecosystem. The results of this study further highlight the notion that the natural variation in pH values^20,21,24^ as well as the interaction of food resource and pH affect organismal trait and performance, and should be considered in further studies on the response of the biota to global change.

## Methods

### Environmental sampling

Environmental conditions were assessed on 12 cruises conducted between May and September of 2015, at a coastal station (1.5 km from the coast, 23°27 S 70°37 W) by measuring temperature, salinity, oxygen, chlorophyll concentration, seawater pH and total alkalinity (Table 1). Temperature, salinity and dissolved oxygen casts were done from just above the bottom (∼ 40 m) to the surface using a calibrated SeaBird SBE19 Plus CTD, equipped with a Wet Star oxygen sensor. During each cruise, 30 L of seawater were obtained with a 10 L Niskin bottle from 10 m depth (Table 1) to provide samples for pH and total alkalinity measurements, as well as to estimate *in situ* ingestion rates (see below) of the copepod *Acartia tonsa*, a neritic (<40 m depth) species with a mean depth of occurrence at 10 m in this upwelling system^31,33^. Water samples for determinations of chlorophyll-*a* concentration (Chl, μg L^−1^), a proxy for phytoplankton abundance, were also collected and then filtered on 200 μm mesh to remove large-sized grazers and debris, but maintaining natural food assemblages. Triplicate samples (200 mL) were filtered onto a GF/F filter (nominal pore size = 0.7 µM) and Chl was extracted for 24 h in 90% acetone v/v and measured in a TD Turner fluorometer^61^.

Temperature-standardized pH (_@25 °C_) was measured in closed 25 mL cells thermostated using a Metrohm 827 pH meter (input resistance, >1 × 1012 Ohm, 0.1 mV sensitivity and nominal resolution at 0.01 pH units) and a glass combined double junction Ag/AgCl electrode (Aquatrode PT1000, N/P 6.0257.000) calibrated with 4 and 7 buffers within 1 h from time of collection. Samples for total alkalinity analysis were collected in borosilicate glass bottles with ground glass stoppers (250 mL) and poisoned with 10 μL HgCl_2_^62^. Total alkalinity (A_T_) was determined using the open-cell titration method^63^, using an automated Alkalinity Titrator AS-ALK2 Apollo SciTech. All samples were analyzed at 25 °C (±0.1 °C) with thermal regulation using a water-bath. The accuracy for A_T_ determinations was controlled against certified reference material (A. Dickson, USA). A_T_ data, temperature, salinity, and pH_@25 °C_ were used to calculate *in situ* pH, *p*CO_2_ and other parameters of the carbonate marine system through the program CO2SYS version 01.05^64^. Thus, *in situ* pH values were reported in accordance to the National Bureau of Standards scale (pH_NBS_) (Guidelines for reporting ocean acidification data in scientific journals, Version 1.0, 2015–03–06). Uncertainties of pH, A_T_ and *p*CO_2_ estimates were 0.03 pH-units, 3 μmol kg^−1^ and 11 μatm, respectively.

### Copepod traits and performance

Plankton samples were collected during the same cruises using a 200 µm WP2 plankton-net equipped with a 1 L non-filtering cod-end, which was hauled vertically from 20 to 10 m depth (Table 1). Within 2 h of collection, undamaged, mature, and visibly healthy adult females of *A*. *tonsa* were sorted under a Leica EZ4HD stereomicroscope, transferred to 300 mL beakers and stored at the same temperature of sampling (14–17 °C) until setting up the experiments. Temperature was adjusted in a cold room whose intra-inter daily thermal variations were ≤0.4 °C.

From copepod samples, up to 40 *A*. *tonsa* females were preserved immediately in 90% ethanol for body length (cephalothorax plus urosome) determinations (mm) under a Leica EZ4HD stereomicroscope. Body length was converted to body mass with the *A*. *clausi* length–dry weight regressions cited by Uye^65^ and to body carbon (BC) assuming that C content was 45% of dry weight^66^. To measure egg production rates (EPR), groups of 25–30 *A*. *tonsa* females were gently pipetted individually into 200 mL closed acid-washed crystallizing dishes filled with natural seawater filtered on 200 μm mesh. Females were incubated at *in situ* temperature and EPR (egg fem^−1^ d^−1^) was the average (±SD) number of eggs produced over 18–20 h^49^. After counting, eggs were preserved (90% ethanol) and the egg diameter (µm) was measured on 20–30 eggs using an inverted microscope Olympus IX-51 within 30 days after preservation. To measure phytoplankton ingestion rates, copepods were pipetted into 660 mL borosilicate acid-washed bottles containing ambient water filled with natural <200 μm food assemblages. Three control bottles without animals and three bottles containing 4–5 adult females of *A*. *tonsa* were placed on a plankton wheel and rotated, end over end at 1.2 rpm and incubated for 24-h at the temperature of copepod collection. Subsamples (200 mL) of control and experimental bottles were filtered in triplicate onto GF/F filter at the beginning and end of the incubation period. Ingestion rates (IR, expressed as µg Chl by female per day), were determined from chlorophyll disappearance during incubations, using the Frost equations^67^, as modified by^68^. While we did not measure pH changes during the copepod incubations, the respiration rate of *A*. *tonsa*^69^ would have accounted for <0.1% of the DIC pool. Thus, changes in pH due to copepods during the incubations were ignored.

Assuming a conservative C:Chl ratio of 50 (since it can reach >100 in the study area^29^), ingestion rates (IR) in μg Chl fem^−1^ d^−1^ were converted to carbon units (μg C). Assuming spherical shaped eggs and a conversion factor of 0.14 × 10^−6^ μg C μm^−3^ ratio^70^, the egg size (diameter) was converted to mass (μg C) and EPR expressed in μg C fem^−1^ d^−1^. Both, IR and EPR, were converted to specific rates by dividing by female body carbon, and the autotrophic egg production efficiency (aEPE) was calculated as the EPR/IR ratio.

### Data analysis

Data pre-processing procedures were provided in Supplementary Information as well as results of Grubb test (S.I. Figure 2). We first employed ordinary single regressions among abiotic variables and copepod traits and performance. Abiotic and biotic relationships were independently explored in distance (temperature, oxygen, salinity, alkalinity, Chl, pH) and similarity (Body size, egg size, EPR, IR) matrices through a Distance based Linear Model (DistLM) which considered a step-wise and Adjusted R^2^ selection procedure and criteria, followed by Principal Coordinate test (PCO). This stepped analysis supported results of single regressions. The relationship between Chl ingestion rate and Chl concentration was determined using ordinary single regression assuming a typical hyperbolically saturating functional response. Based upon the inflection point from the functional response, Chl concentration was categorized as either high (H >1 µg Chl L^−1^) or low (L <1 µg Chl L^−1^) levels. The threshold between present day (≤400 µatm) and future (>400 µatm) *p*CO_2_ ocean conditions was superimposed on *p*CO_2_ concentrations estimated at 10 m depth in the study site, and the equivalent pH levels were indicative of “High” (pH > 7.89) or low (pH < 7.89) conditions. Upwelling pH levels were compared (Kruskal-Wallis test) to pH values globally considered in laboratory studies (*n* = 40) assessing copepod sensitivity to OA conditions expected by the years 2100 and 2300^40^. Upwelling pH values were significantly higher than both OA scenarios, although low pH values observed occasionally at 10 m depth in the upwelling site overlapped those of the 2100 OA condition. Copepod traits and performance were thus compared among the categorized Chl-a/pH levels (H/H, H/L, L/H and L/L) by a 2-factor (Chl + pH) permutational analysis of variance (PERMANOVA). On this design, H and L Chl treatments were specifically contrasted through pair-wise comparison tests within H and L pH treatments. All PERMANOVA tests were preceded by PERMDISP tests to verify the assumption of homogeneity of multivariate dispersions. Statistical analyses were performed in PRIMER6^+^.

## Data Availability

The dataset generated during the current study will be available on an online repository (PANGAEA), and it is available from the corresponding author as well.
